# Supplementary material for: Impact of Pelvic Fracture Sites on Fibrinogen Depletion in Patients with Blunt Trauma: A Single-Center Cohort Study
Source: J Clin Med. 2022 Aug 11;11(16):4689. doi: 10.3390/jcm11164689 (PMC9409758; doi:10.3390/jcm11164689)
Supplement: Supplementary file 1 [file jcm-11-04689-s001.zip › jcm-1807243-supplementary.pdf]

Supplementary Table S1. Results of univariable and multivariable linear regression analyses assessing pelvic fracture site on minimum fibrinogen level within 24 hours from arrival with WSES classification as a covariate.

	Univariable analysis			Multivariable analysis		
	Estimate	SE	P value	Estimate	SE	P value
Pelvic fracture site						
Ilium	9.3	16.0	0.563	18.2	16.7	0.277
Pubis	-23.7	11.1	0.035	0.5	13.3	0.969
Ischium	-20.3	11.8	0.088	-13.6	13.1	0.300
Acetabulum	-7.7	13.8	0.577	2.6	12.9	0.841
Sacrum	-37.1	11.1	0.001	-25.7	12.7	0.045
Sacroiliac joint diastasis	-34.0	17.2	0.051	2.3	21.6	0.914
Pubic symphysis diastasis	-37.7	52.1	0.470	4.4	57.7	0.939
Concomitant injury						
Head	-27.8	16.5	0.095	-39.0	18.6	0.038
Thorax	-45.6	15.8	0.005	-17.6	16.5	0.288
Abdomen	-27.9	18.6	0.137	-19.5	18.6	0.296
Age	0.0	0.4	0.997	0.1	0.4	0.846
WSES classification	-29.0	6.7	<0.001	-24.2	8.3	0.004

SE standard error; *WSES* World Society of Emergency Surgery
